# Novel compound heterozygous mutations of DOCK2 associated with early-onset recurrent infections

**DOI:** 10.3389/fimmu.2026.1830547

**Published:** 2026-10-26

**Authors:** Zhi Yang, Linlin Wang, Yang Wang, Xiaona Zhu, Yanyan Huang, Ying Luo, Jun Yang, Tingyan He

**Affiliations:** 1 Department of Rheumatology and Immunology, Shenzhen Children’s Hospital, Shenzhen, China; 2 Department of Pediatrics, Baiyun Hospital Affiliated to Guizhou Medical University, Guiyang, China

**Keywords:** DOCK2, HLH, immunodeficiency, inborn error of immunity, ITp

## Abstract

**Introduction:**

Dedicator of cytokinesis 2 (DOCK2) deficiency is a rare combined immunodeficiency characterized by defects in both cellular and humoral immunity and caused by pathogenic mutations in the DOCK2 gene. Herein, we report a 3-year-8-month-old Chinese girl presenting with recurrent infections, immune thrombocytopenia (ITP), elevated IgE levels, and a hemophagocytic lymphohistiocytosis (HLH)-like episode to broaden the clinical phenotype spectrum of the DOCK2 defect.

**Methods:**

All tests and molecular genetic analyses were performed after obtaining informed consent from the parents. Demographic, clinical, and laboratory findings were abstracted from outpatient and inpatient encounters. Whole-exome sequencing (WES) was performed on peripheral blood cells to identify the genetic etiology, followed by immunological evaluation (including lymphocyte subset analysis, T-cell receptor Vβ repertoire analysis, and neutrophil oxidative function assays) and in silico prediction. Sanger sequencing was used to confirm the identified variants.

**Results:**

WES identified two novel compound heterozygous variants in DOCK2 (c. 106 C>T, p.R36X and c. 2150T>C, p.L717P), each inherited from one parent. Immunological evaluation revealed combined immune defects involving T cells and B cells, including reduced T/B cell subsets, skewed TCR repertoire, and impaired T cell proliferation. In silico analyses indicated that the variants affect highly conserved residues and are predicted to disrupt protein structure. The patient received supportive treatment, regular intravenous immunoglobulin replacement therapy, and sulfonamide prophylaxis, resulting in effective disease control.

**Discussion:**

This case expands the genotypic and phenotypic spectrum of DOCK2 deficiency and highlights its critical role in human immune regulation. It also underscores the importance of early genetic diagnosis to guide timely clinical management. However, functional validation experiments were not performed to further confirm the pathogenicity of the identified variants, and additional protein expression studies are warranted.

## Introduction

Dedicator of cytokinesis 2 (DOCK2) is the second DOCK protein identified in mammals and a member of the DOCK-A subfamily. It functions as a major guanine nucleotide exchange factor (GEF) that regulates small Rho GTPases, particularly Rac (1). DOCK2 is predominantly expressed in hematopoietic and lymphoid tissues and mediates the GDP–GTP exchange of Rac through its DHR-2 domain. Upon activation, Rac acts downstream of G protein–coupled receptors (GPCRs), as well as antigen and complement receptors, and plays a crucial role in actin cytoskeleton remodeling. The activated Rac is involved in a wide range of immune-related cellular processes, including lymphocyte trafficking, neutrophil chemotaxis, reactive oxygen species (ROS) generation, production of type I interferons and TNF-α, and immunological synapse formation (1–3).

In 2015, Dobbs et al. first described five unrelated patients presenting with early-onset, severe viral and bacterial infections caused by biallelic loss-of-function (LOF) variants in *DOCK2* (4). DOCK2 deficiency leads to profound immune dysfunction, affecting multiple leukocyte populations. Reported immunological abnormalities include impaired T- and B-cell chemotaxis and activation, defective NK cell degranulation, impaired cytoskeletal rearrangement, reduced neutrophil ROS production, and compromised type I interferon responses in peripheral blood mononuclear cells (1, 4–7). Clinically, affected individuals typically present with early-onset, recurrent, and invasive infections accompanied by T- and B-cell lymphopenia, consistent with the characteristics of combined immunodeficiency disease (CID). Additional manifestations reported in DOCK2-deficient patients include chronic diarrhea, thrombocytopenia, hemophagocytic lymphohistiocytosis (HLH), and seizures (4, 6, 8–11). In the absence of Hematopoietic Stem Cell Transplantation (HSCT), patients with DOCK2 deficiency succumb to recurrent respiratory and gastrointestinal infections at an early age (4, 6, 11). To date, like other newly identified inborn errors of immunity (IEIs), the clinical spectrum and underlying pathophysiological mechanisms of DOCK2 deficiency remain incompletely characterized.

In this study, we describe the clinical manifestations and immunological phenotype of a Chinese girl with novel compound heterozygous variants in *DOCK2*, thereby further expanding the phenotypic and genetic spectrum of DOCK2 deficiency.

## Methods

### Patients and control subjects

Diagnosis of DOCK2 deficiency was based on clinical features, whole exome sequencing (WES), and functional assessments of immune cells. The study was conducted in accordance with the Declaration of Helsinki. The study was approved by the Ethics Committee of the Shenzhen Children’s Hospital. The healthy control samples for this study were derived from healthy children without personal or family history of genetic or immunological disorders. All study participants and healthy controls (HC), or their guardians, provided written informed consent to participate.

### Genetic diagnosis

WES Genomic DNA was extracted from peripheral blood cells isolated from the patient and her parents. The exonic regions and flanking splicing or intronic junctions of the whole genome were captured and sequenced using an Illumina HiSeq 2000 sequencer conducted by MyGenostics (Beijing, China). The FASTQ files were mapped to the human reference genome (hg19). The functional effects of variants were predicted using three algorithms (PolyPhen-2, SIFT, and VariantTaster), and amino acid conservation among species was analyzed. Sanger sequencing was used to confirm pathogenic variants.

### Epinephrine test

As a functional readout of immune cell trafficking, peripheral blood leukocyte mobilization in response to epinephrine was assessed. The specific protocol are as follows: a subcutaneous injection of 0.1 mL of 1% epinephrine solution was administered. Peripheral leukocyte counts and differential analyses were performed before injection, at 5, 10, 15, and 30-minutes following post administration.

### Intracellular production of hydrogen peroxide

Hydrogen peroxide (H_2_O_2_) production by neutrophils was assessed using dihydrorhodamine (DHR)-1, 2, 3 flow cytometry (12). Briefly, heparinized peripheral blood samples from the patient or health controls were incubated with phorbol 12-myristate 13-acetate (PMA; Sigma, USA) and DHR(Sigma, USA)(the stimulated tube) or DHR only (the unstimulated tube) for 20 min at 37°C. Subsequently, erythrocytes were lysed by incubation with an erythrocyte lysis solution (TIANGEN, China) for 15 min at room temperature. Then samples were centrifuged at 360g for 5 min and washed twice with PBS. Finally, the cells were analyzed using a FACS Canto II flow cytometer. The stimulation index (SI) was calculated as the ratio of the geometric mean fluorescence intensity (MFI) of PMA-stimulated neutrophils to that of unstimulated neutrophils. The SI and percentage of DHR+ neutrophil populations were analyzed.

### Lymphocyte proliferation test

Peripheral blood mononuclear cells (PBMCs) were isolated from whole blood of the patient and controls, re-suspended in PBS containing carboxyfluorescein succinimidylester (CFSE, Invitrogen, USA) and incubated at 37°C for 10 min. The labeling reaction was quenched with 10 ml RPMI 1640 containing 10% FBS, and the cells were washed twice with the same culture medium. The cells were re-suspended and cultured in 96-well plates with 5 ug/mL phytohemagglutinin (PHA, Sigma, USA) or 1 ug/mL anti-human CD3/CD28 antibodies (Biolegend, USA) for 72 h at 37 °C in a humidified atmosphere containing 5% CO2. Subsequently, the cells were stained with CD4-APC and CD8-PE antibodies (Biolegend, USA). T-cell proliferation was assessed by flow cytometry(FCM).

### CDR3 spectra typing

Each T cell receptor (TCR) Vβ fragment was amplified by PCR using one of 23 Vβ-specific primers and in combination with a 5′ FAM-labeled Cβ primer, as previously described (13). The PCR products were sequenced by Sangong Biotechnology Co. (Shanghai, China). Data analysis was performed using GeneMapper version 3.5(Applied Biosystems, USA). TCR Vβ diversity was evaluated using a scoring system, as described previously (14) (complexity score = (sum of all the peak heights/sum of the major peak heights) × number of major peaks. Major peaks were defined as those with amplitudes of at least 10% of the sum of all the peak heights). TCR Vβ subfamilies with complexity score less than 4 were considered to have skewed CDR length distribution.

### Analysis of the proportion of Th1/2/17 subsets and lymphocyte immunophenotyping

For Th1/2/17 subsets analysis, PBMCs were stimulated with Cell Stimulation and Protein Transport Inhibitor Cocktail(containing brefeldin A, ionomycin, monensin, and PMA, Biogems, USA) for 4 h at 37 °C. Cells were washed twice with PBS and stained with CD3-PerCP-Cy5.5 and CD8-BV510 (BD Biosciences, USA) for 30 min at room temperature. After two additional washes with PBS, cells were fixed with Fixation and Permeabilization Solutions (BD Biosciences, USA) for 30 min at 4 °C, and subsequently permeabilized with Perm/Wash Buffer (BD Biosciences, USA) for 20 min at room temperature. The cells were then incubated with IFN-γ-APC, IL-4-AF88, and IL-17A-PE (BD Biosciences, USA) for 30 min. to allow intracellular staining. The proportions of cytokine-producing cells were analyzed on a FACSCanto II flow cytometer.

Detailed lymphocyte subset analysis (T, B, NK cells and their subpopulations) was performed according to the reference ranges established by Zhao et al. for healthy Chinese children (15).Immunophenotyping of lymphocyte subpopulations was performed with the following antibodies: CD3-PerCP, CD4-FITC, CD8-BV510 CD45RA-PE-Cy7, CD27-APC, TCR aβ-PE, TCR γδ-BV421, CD19-APC, CD27-V450, IgD-BV510, CD24-PE, and CD38-PerCP (BD Biosciences, USA). The samples were acquired on a FACSCanto II flow cytometer, and the data were analyzed using FlowJo (BD Bioscience, USA).

## Results

### Clinical manifestations of the DOCK2-deficient patient

The patient was a 3-year-8-month-old girl born to a non-consanguineous family in China. She was delivered at term with a birth weight of 3500 g and received routine childhood vaccinations, including Bacillus Calmette–Guérin (BCG), Hepatitis B, Poliomyelitis, Diphtheria, Pertussis, Tetanus, Measles, Mumps, Rubella, Meningococcus, Japanese Encephalitis, Varicella Zoster, Hepatitis A, Hemophilus Influenzae type B, Enterovirus Type 71, and Influenza. She had an older sister who died at 2.3 years of age due to severe infection complicated by acute renal failure, and a currently healthy older brother (**Figure 1A**).

**Figure 1:**
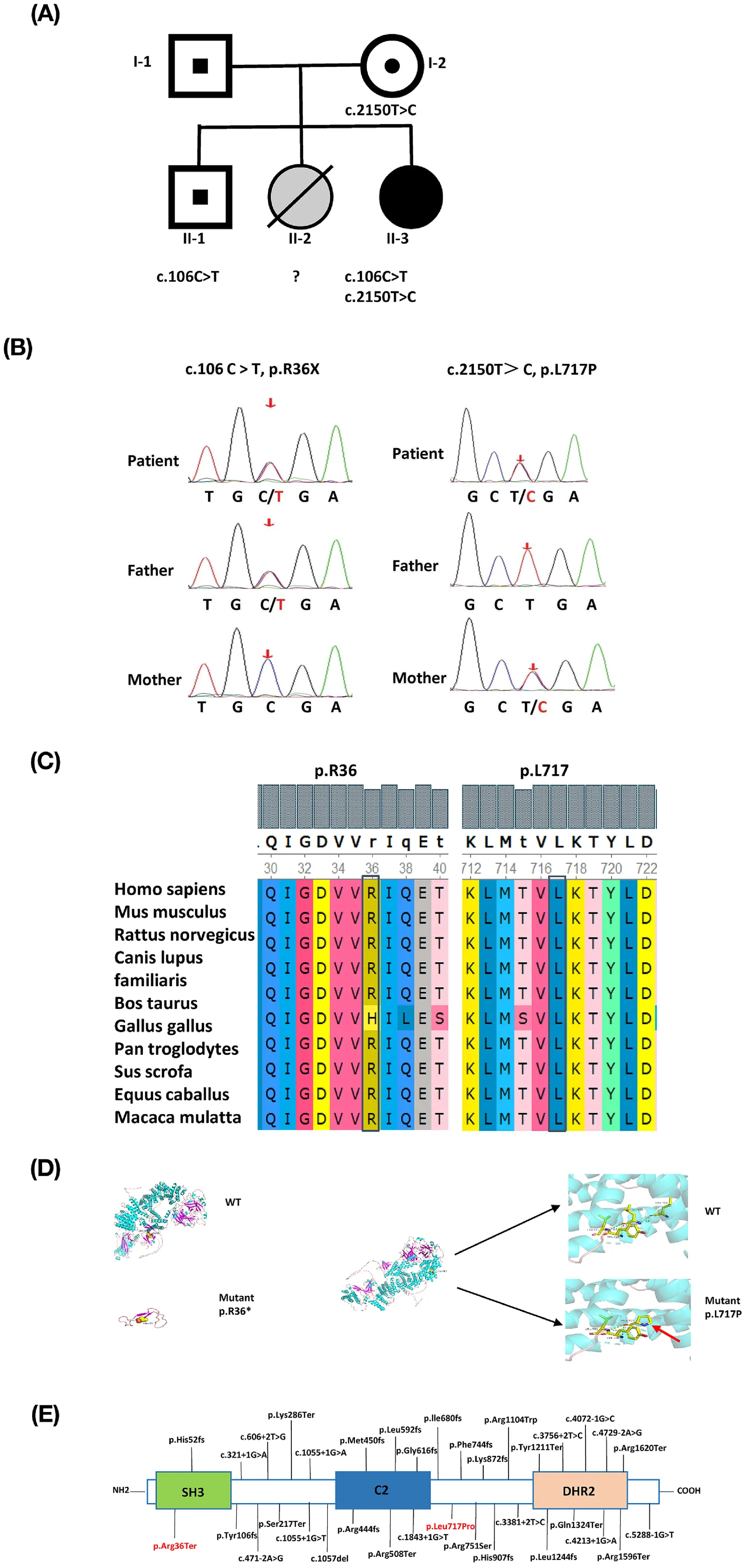
The family pedigree and genetic analysis of the patient. **(A)** Circles represent female family members, and squares represent male family members. White symbols indicate healthy individuals, grey symbols indicate possible affected individuals, and black symbols indicate the proband. **(B)** The identified *DOCK2* variants in the patient and family members are shown and were confirmed by Sanger sequencing. **(C)** Conservation analysis of the c.106C>T, p.R36X variants across multiple species. **(D)** Predicted effects of the two variants on the protein structure based on structural modeling. **(E)** Schematic representation of the identified *DOCK2* variants relative to the protein structure, showing the SRC homology 3 (SH3), DOCK homology region 1 (DHR-1), and DOCK homology region 2 (DHR-2) domains. The mutations identified in our patient are indicated by red dots.

At 7 months of age, she presented with fever and cough, and her chest computed tomography revealed diffuse interstitial pneumonia. Laboratory investigations demonstrated elevated ferritin levels, cytopenia, hemophagocytosis, and negative pathogen testing. This was an HLH-like episode, and the patient recovered following anti-infective treatment and intravenous immunoglobulin (IVIG) therapy.

At 1.8 years of age, the patient developed hand-foot-and-mouth disease complicated by immune thrombocytopenia (ITP). Thereafter, laboratory evaluations frequently revealed lymphopenia and granulocytopenia. Between 2.3 and 3.4 years of age, she experienced five recurrent episodes of pneumonia or bronchitis, with identified pathogens including parainfluenza, Influenza A virus, respiratory syncytial virus, Hemophilus influenzae, Bocavirus, streptococcus pneumoniae, and human metapneumovirus. In addition, she developed mumps twice and Naso sinusitis once. Subsequently, she was hospitalized on three occasions for recurrent fever, cough, and rhinorrhea lasting more than two months. Although her symptoms temporarily improved, they relapsed after discontinuation of antibiotic therapy. She was therefore started on regular IVIG replacement therapy combined with sulfonamide prophylaxis, after which no further infections were observed at the most recent follow-up.

### The compound variants in *DOCK2*

WES revealed compound heterozygous variants in *DOCK2* (OMIM: 603122), including c.106 C>T, p.Arg36Ter and c.2150T>C, p.Leu717Pro (**Figures 1B, E**), which were inherited from her father and mother, respectively. The predicted scores for p.R36X using Variant Taster and GERP++ were 1 and 3.98, respectively, indicating disease-causing effects and high evolutionary conservation (**Figure 1C**). For p.L717P, the predicted scores from SIFT, PolyPhen_2, Variant Taster, and GERP++ were 0, 0.998, 1, and 5.58, respectively, suggesting a damaging or probably damaging effect, disease causality, and high conservation (**Figure 1C**). Both variants were further confirmed by Sanger sequencing (**Figure 1B**). Structural modeling predicted that these variants would alter protein conformation (**Figure 1D**). No pathogenic variants were identified in other genes associated with recurrent infections, HLH, myelodysplastic dysplasia, granulocytopenia, lymphocytopenia, or thrombocytopenia. We further performed qPCR to assess DOCK2 transcript levels in patient-derived peripheral blood mononuclear cells (PBMCs). However, we observed no significant difference in DOCK2 mRNA expression between the patient and healthy controls (**Supplementary Figure 2**).

### Disruption of T/B cell subsets and skewed TCR repertoire

The patient’s lymphocyte count was below the normal range count (**Figure 2A**). We analyzed the lymphocyte subsets of the patient by FCM. The patient exhibited decreased numbers of total lymphocytes, CD4^+^ T cells, and CD8^+^ T cells, with most T-cell subsets also reduced (**Table 1**). Additionally, the numbers of B cells subtypes were lower than those in age-matched controls (**Table 1**). The patient’s TCR-Vβ subfamilies revealed monoclonal or oligoclonal peaks (**Figure 2B**). Following stimulation with PMA and ionomycin, the percentage of interferon-gamma-producing Th1 cells in the patient was lower than that in health controls(**Figure 2C**). Furthermore, T cell proliferation was impaired in the patient (**Figure 2D**).

**Table 1:** Immunological profile of a patient with novel DOCK2 compound heterozygous variants.

Lymphocyte subsets	Value (cells/uL)	Reference range^1^(cells/uL)	Immunoglobulins and cytokines	Value	Reference range^2^
T cell	271.04↓	1775-3953	IgG(g/L)	8.6	
CD8	118.72↓	531-1521	IgG1(g/L)	7.25	3.20-10
CD8 naïve	45.71↓	295-971	IgG2(g/L)	1.88	0.63-3.0
CD8 CM	50.10↓	54-379	IgG3(g/L)	1.19	0.14-1.33
CD8 EM	8.11	4-163	IgG4(g/L)	0.01	0.03-1.27
CD8 TEMRA	14.84	3-294	IgM(g/L)	1.17	0.40-1.50
CD4	95.14↓	948-2477	IgA(g/L)	0.44	0.30-1.50
CD4 naïve	10.66↓	530-1837	IgE(g/L)	8805↑	<60
CD4 CM	73.06↓	220-800	C3(g/L)	1.14	0.83-1.52
CD4 EM	10.75↓	14-93	C4(g/L)	0.3	0.13-0.37
CD4 TEMRA	0.64	0-12	IL-2(pg/mL)	0	0-6.64
DNT	0.89↓	16-58	IL-4(pg/mL)	1.47	0-4.19
γδT	89.99↓	128-520	IL-6(pg/mL)	13.1	0-11.09
B	269.28↓	537-1464	IL-10(pg/mL)	10.97	0-5.61
naïve B	202.77↓	371-1197	TNF-α(pg/mL)	2.46	0-6.01
memory B	7.08↓	33-146	IFN-γ(pg/mL)	4.48	0-4.43
transitional B	12.66↓	33-181			
plasmablast	4.17	4-88			
NK	274.56	241-978			

^1^Date from Reference values for peripheral blood lymphocyte subsets of healthy children in China; ^2^Data from department of Laboratory Medicine, Shenzhen Children’s Hospital.

**Figure 2:**
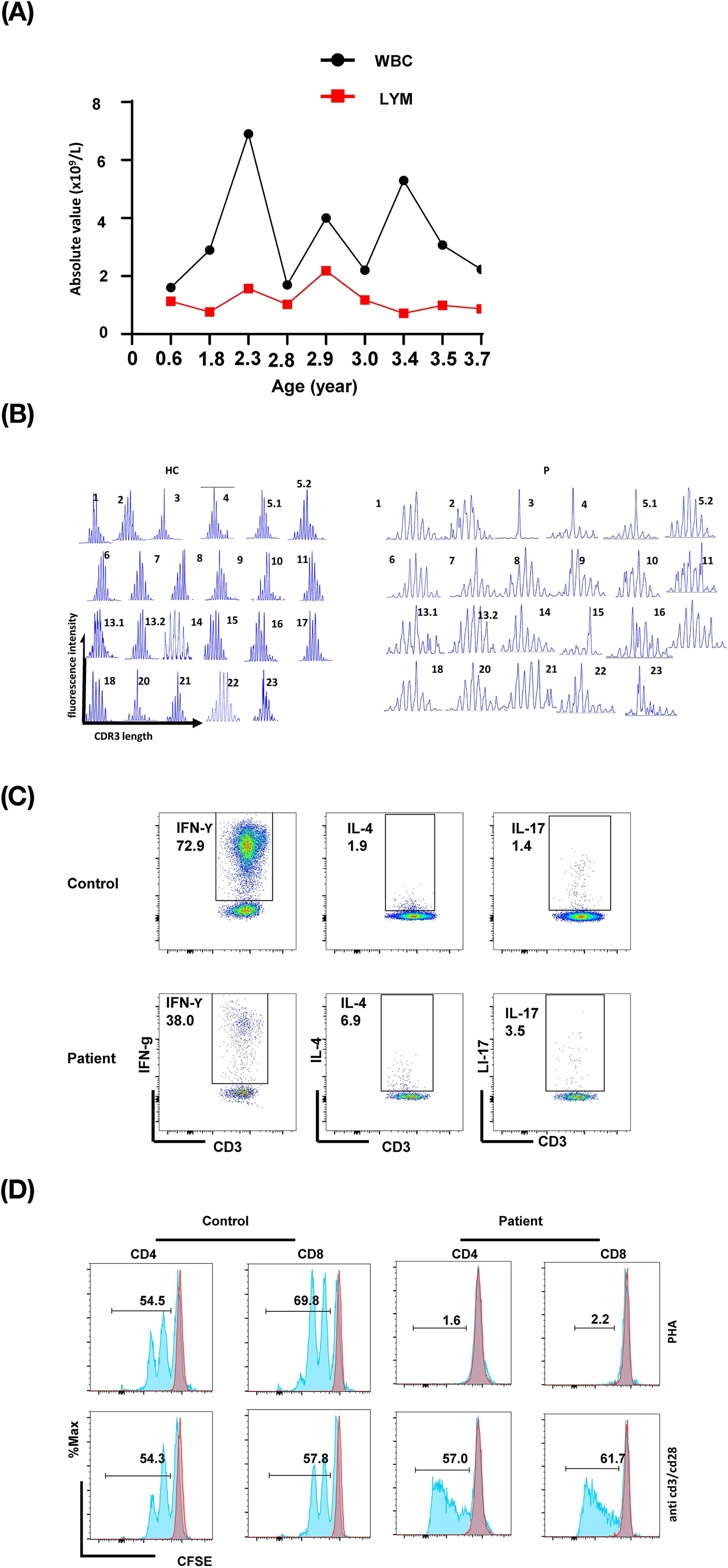
Disruption of T cell subsets associated with DOCK2 deficiency. **(A)** White blood cell and lymphocyte count of the patient with DOCK2 deficiency; **(B)** CDR3 size distribution of TCR-Vβ subfamilies in the patient compared with a healthy control; **(C)** Percentages of Th1 and Th2 cells in the patient compared with a healthy control by flow cytometry; **(D)** Proliferation of CD4^+^ and CD8^+^ T cells in the patient compared with a healthy control by flow cytometry.

### Disorders of humoral immunity and serum cytokine

The patient’s serum total IgG, IgM, and IgA levels were within the normal range. The total IgE level was markedly elevated at 8805 IU/ml. Additionally, the patient showed increased levels of serum IL-6, IL-10, and IFN-γ (**Table 1**).

### Neutrophil in DOCK2-deficient patient

The patient’s neutrophil count was below the normal range (**Figure 3A**). Following subcutaneous injection of epinephrine, the counts of neutrophil increased to more than twice the baseline level (**Figure 3B**). Neutrophil oxidative function was assessed using DHR assay. The patient’s neutrophils showed 85.3% DHR^+^ cells with a stimulation index (SI) of 56.9, whereas the controls exhibited 95.3% DHR^+^ cells with an SI of 95.3. The respiratory burst index of DOCK2 patients is lower than that of the healthy control group, but it is within the normal range (**Figure 3C**).

**Figure 3:**
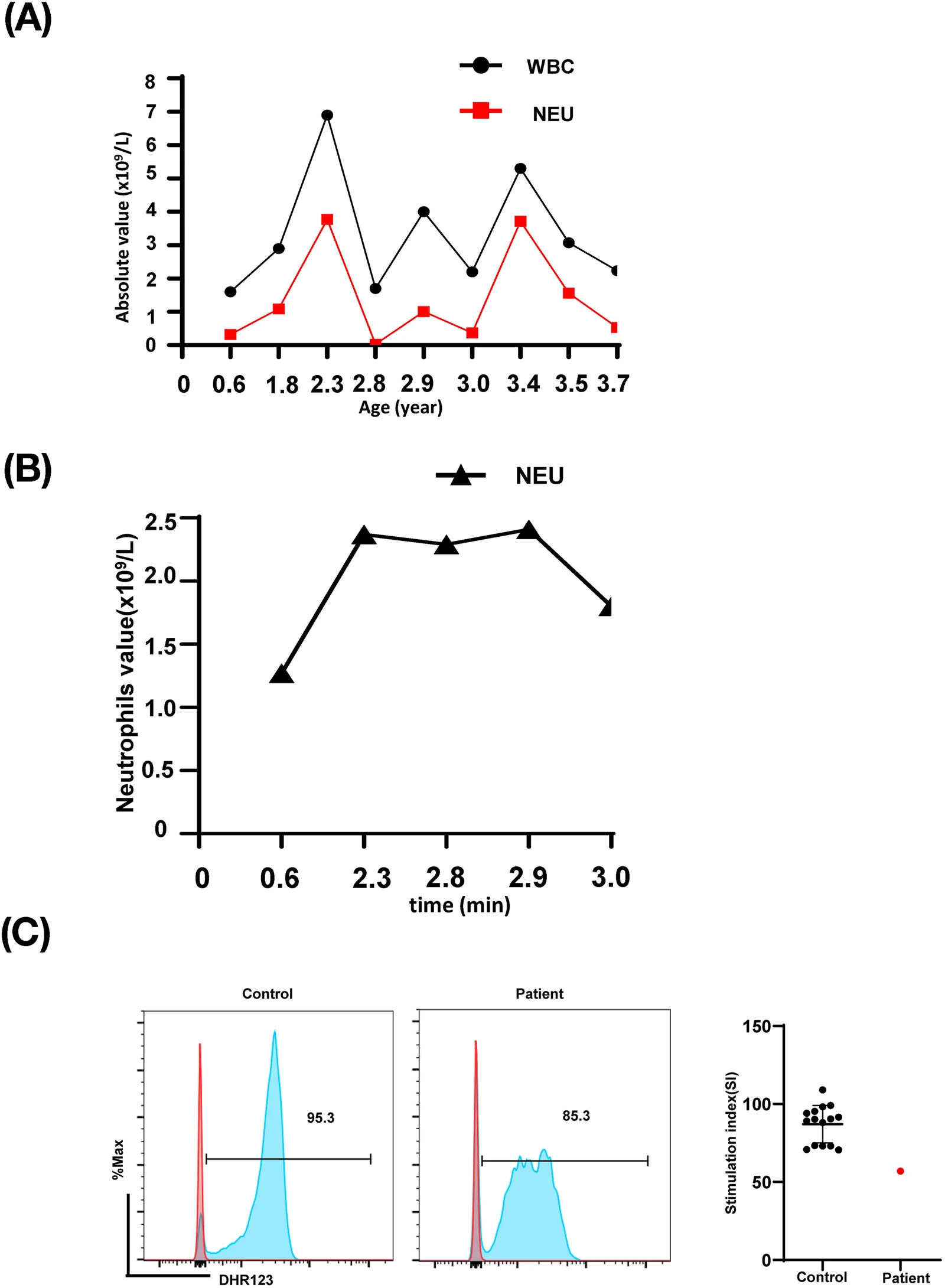
The disruption of neutrophils in the patient with DOCK2 deficiency. **(A)** White blood cell and neutrophil counts of the patient with DOCK2 deficiency; **(B)** Neutrophil counts of the patient following subcutaneous epinephrine injection; **(C)** Neutrophil respiratory burst measured by DHR-123 oxidation (Left)Figure. The median fluorescence intensity of gated neutrophils and the mean ± SD stimulation index (SI) are shown (Right).

## Discussion

In this study, we reported a 3-year-and-8-month-old girl presenting with recurrent, early-onset infections, although she did not experience severe and life-threatening infections. Granulocytopenia and lymphocytopenia were frequently observed during episodes of illness. The patient developed immune thrombocytopenia during a hand-foot-and-mouth infection. The patient also presented with a HLH-like episode, which had been reported in previous cases (6, 8, 9). Sakai et al. described that although DOCK2^−/−^NK cells can effectively bind to target cells *in vivo*, their cytotoxicity and synapse formation were significantly impaired (8). Given that HLH itself is known to induce secondary suppression of NK cell activity, the absence of acute-phase functional data in our case makes it difficult to distinguish between a primary DOCK2-driven defect and a secondary HLH-mediated inhibitory effect. However, based on the established molecular consequences of DOCK2 loss, we postulate that the underlying NK cell dysfunction, compounded by the systemic inflammatory milieu, may have collectively contributed to the HLH-like manifestations observed in our patient. Future prospective evaluations of NK cell function at the time of active inflammation would be invaluable to further disentangle these pathogenic contributions.

DOCK2 deficiency impairs T cell development and function (16). In our patient, both CD4^+^ and CD8^+^ T cells were significantly reduced, including their naïve subsets, and TCR diversity is mildly skewed, indicating defective T cell maturation; however, statistical comparisons with healthy controls could not be performed due to the lack of a sufficiently sized age-matched control group, and these findings should therefore be interpreted as descriptive observations. Mechanistically, DOCK2 is essential for lymphocyte chemotaxis: DOCK2-deficient mice exhibit migration defects of T and B cells in response to chemokines, leading to lymphocytopenia, lymphoid follicle atrophy, and loss of marginal-zone B cells (4, 16). At the cellular level, DOCK2 deficiency abolishes chemokine-induced Rac activation and actin polymerization, reduces WASP activation, accelerates its degradation, and diminishes actin accumulation in early-activated T cells (16). Antigen-induced TCR and lipid raft translocation to the APC interface is severely impaired, reducing antigen-specific T cell proliferation, while both positive and negative thymic selection are compromised (17). In addition, the efficacy of both positive and negative selection was reduced in DOCK2-deficient mice (17). These findings indicate that DOCK2 may regulate T cell responsiveness primarily through Rac-mediated cytoskeletal remodeling.

B cell abnormalities frequently accompany T cell defects in DOCK2 deficiency (4, 6). In affected patients, B cell counts and IgG levels range from low to normal, while IgM can be decreased or elevated (4, 18, 19). Our patient showed reduced CD19^+^ B cells across naïve, memory, and transitional subsets. Total IgG, IgM, and IgA remained within the normal range, and serum IgE was markedly elevated, Both CD4^+^ and CD8^+^ T cells were significantly reduced, with reduced Th1 cell function and a relative skewing toward Th2 responses. Mechanistically, BCR-mediated Rac activation is nearly abolished in DOCK2-deficient B cells, resulting in impaired B cell spreading and defective BCR micro-cluster formation (1, 2, 20). Conditional knockout mouse models further show that DOCK2 expression in B cells is essential for antigen-specific IgG responses, highlighting the role of the DOCK2–Rac axis in plasma cell differentiation and IgG production (20). Although most patients maintain normal total IgG, IgM, and IgA levels, antibody function may be compromised, consistent with our patient’s hyper-IgE. By analogy with DOCK8 deficiency and CDC42 defects, DOCK2 deficiency may similarly lead to hyper-IgE, likely exacerbated by Th2-biased immunity (5, 21). Together with the occurrence of ITP, these findings indicate impaired B cell function, likely affecting class switching and overall humoral immunity.

During inflammation, neutrophils are the first type of innate immune cells that migrate to the sites of infection. Neutrophils recognize and engulf microorganisms and kill them by releasing mediators such as reactive oxygen species (ROS) (22, 23). Neutrophil chemotaxis and ROS production depend on Rac small GTPase activation, which is severely impaired in DOCK2 deficiency (23, 24). Study by Moens et al. demonstrates that DOCK2-deficient neutrophils exhibit partially impaired cytoskeletal rearrangement and reactive oxygen species (ROS) production in human patients (7). In our patient, neutrophil counts were reduced during acute infections but could return to normal upon recovery, and significantly increased after subcutaneous adrenaline injection. In addition, the respiratory burst index in our DOCK2 patient was lower than that of the healthy control, but it is within the normal range. However, owing to technical constraints, we did not perform additional functional validation assays on neutrophils.

Next-generation sequencing identified novel compound heterozygous variants in DOCK2. One variant, c.106C>T, introduced a premature stop codon (P.R36X), resulting in protein truncation, while the other, c.2150T>C, caused a P.L717P substitution. Neither of the two genetic variants has a population frequency reported in gnomAD. According to the ACMG guidelines, both variants are classified as likely pathogenic variants. Both sites are highly conserved across species, and structural modeling predicted that these variants would alter protein conformation. The missense variant (c.2150T>C) may act as a hypomorphic allele, retaining partial protein function, which is consistent with the patient’s recurrent but non-lethal infections. Although functional validation was not performed, the patient exhibits combined immune defects affecting T cells, B cells, and possibly NK cells, supporting a high likelihood that these two variants may be pathogenic.

This study has several limitations. First, functional validation experiments were not performed to further confirm the pathogenicity and biological effects of the identified *DOCK2* variants. In addition, due to the young age of the patient, recruiting age-matched healthy controls was challenging, resulting in a limited number of controls enrolled during the same period to perform meaningful statistical comparisons.

## Conclusion

We report a patient with novel compound heterozygous DOCK2 variants presenting with recurrent infections, ITP, and HLH-like episodes, accompanied by combined immune defects. These findings expand the genotypic and phenotypic spectrum of DOCK2 deficiency, underscore the critical role of DOCK2 in human immune regulation, and highlight the importance of early genetic diagnosis to guide timely clinical management.

A major limitation of this study should be acknowledged. Owing to the limited volume of peripheral blood obtainable from the young patient and the parents’ refusal to provide additional samples for research, we were unable to perform protein-level analyses or comprehensive functional assays to directly validate the pathogenicity of the compound heterozygous mutations. Consequently, while the genetic analysis and in silico predictions strongly suggest that these variants are likely pathogenic, we cannot currently establish a definitive causal relationship. Thus, at this stage, we consider these compound heterozygous mutations potentially pathogenic, and further protein expression studies and functional experiments are warranted to confirm their role in the disease.

## Data Availability

The raw sequence data presented in this study have been deposited in the NCBI Sequence Read Archive (SRA) under BioProject accession number PRJNA1542262.
