# Supplementary material for: Novel compound heterozygous mutations of DOCK2 associated with early-onset recurrent infections
Source: Front Immunol. 2026 Oct 26;17:1830547. doi: 10.3389/fimmu.2026.1830547 (PMC13745346; doi:10.3389/fimmu.2026.1830547)
Supplement: Supplementary file 1 [file DataSheet1.docx]

RNA analysis

Peripheral blood mononuclear cells (PBMC) were prepared by density gradient centrifugation using Lymphocyte Separation Media (MP Biomedicals, USA) from the patient and normal control subject. Total RNA was extracted from PBMC using an RNAqueous Kit (ThermoFisher, USA). DOCK2 cDNA from exon2 to exon22 (NCBI Reference Sequence: NM_004946.3) was synthesized and amplified using SuperScript III One- Step RT-PCR System with Platinum Taq DNA Polymerase kit (ThermoFisher, USA) according to the manufacturer’s protocol. The forward primer was 5’-CTGGCGCAAAGCTGACAAG-3′ and the reverse primer was 5’-TCTTTGCCTTCATAAAGCTGTGA-3′. The target production length was 2277 bp and sequenced by Sanger method (Sangong Biotechnology Co, China). The long PCR product sequencing results showed that the patient’s mRNA of DOCK2 from exon2 to exon22 was entirely transcribed, with two heterozygous variants (c.106 C>T and c.2150T>C).

**Supplementary Figure 1** Sequencing PCR product of DOCK2 from exon2 to exon22 of the patient.

Then, we further confirmed the mRNA transcriptions of exon2 and exon22 of DOCK2 separately by PCR, using Premix Ex Taq DNA Polymerase Hot Start Version (Takara, Japan), with the following primers:

2-F: 5’-CTGGCGCAAAGCTGACAAG-3’,

2-R: 5’-TGCCCTGTAACATTTTGTGCT-3’(target length: 164bp);

22-F: 5’-CCAACAGCATTTCAGTGCGA-3’,

22-R:5’-ACTCCATCTGTTCTTTGCCTTCA-3’ (target length 197bp).

Then the PCR products were sequenced by Sanger method. The PCR and sequencing results showed again that exon2 and exon22 of DOCK2 from the patient were successfully transcribed, with two heterozygous variants (c.106 C>T and c.2150T>C).

Finally, we detected mRNA expression of DOCK2 by Real-Time Quantitative PCR. Total RNA was extracted from PBMC using RNAqueous Total RNA Isolation Kit (ThermoFisher Scientific, USA) from the patient and 3 unrelated health controls. The cDNAs were transcribed using transcriptor first strand cDNA synthesis kit (Roche, USA) and then quantified by RT-PCR using BrightCycle Universal SYBR Green qPCR Mix with UDG (ABcloncal, China) on an LightCycler 480 Instrument (Roche, USA). The thermal cycling conditions were: 37°C for 2min, 95 °C for 3 min denaturation, followed by 40 cycles of 95 °C for 5s and 60 °C for 30s. β-Actin was used as a normalization control. The 22-F and 22-R were used as the primers. The relative expression levels of DOCK2 were analyzed with the 2^−ΔΔCt^ method.

**Supplementary Figure 2** Relative mRNA expressions of DOCK2 from the patient and 3 health controls by RT-PCR
